# Experiences in COVID-19 clinical management and health-care pathways in the Western Pacific

**DOI:** 10.5365/wpsar.2023.14.5.1017

**Published:** 2023-06-22

**Authors:** Saho Takaya, Ji Young Lee, Takeshi Nishijima, Masahiro Zakoji, Howard L Sobel

## Abstract

The coronavirus disease (COVID-19) pandemic has transformed clinical practice and health systems. This paper provides an overview of COVID-19 clinical management and health-care pathway challenges that the World Health Organization and its Member States in the Western Pacific Region have faced. The experiences and lessons identified can help countries to better prepare for future pandemics.

The coronavirus disease (COVID-19) pandemic has highlighted the importance of optimizing clinical management and health-care pathways during public health emergencies. This report provides an overview of clinical management and health-care pathway challenges that the World Health Organization (WHO) and its Member States in the Western Pacific Region faced during the COVID-19 pandemic.

On 31 December 2019, the WHO Representative Office for China notified the Regional Office for the Western Pacific that cases of pneumonia of unknown origin had been reported in Wuhan, Hubei Province. (*1*) Since then, health-care workers have had to adapt their approach to clinical management and health-care pathways as they tackled multiple challenges caused by unprecedented case numbers, including overwhelmed hospitals, inadequate bed capacity and resources, and staff shortages as they too contracted COVID-19. Moreover, as new evidence emerged, health-care workers were constantly having to make adjustments to their clinical practice and care pathways. Many health systems around the world struggled to provide the right care to the right patients at the right time while safeguarding wider essential health services.

In the early phase of the pandemic, patient flow in hospitals was compromised by the requirement of a negative polymerase chain reaction (PCR) test and clinical recovery for releasing patients from isolation. (*2*) This meant that asymptomatic patients remained in isolation long after they were no longer infectious, taking up vital hospital bed capacity. Although test-based criteria were changed to time-based criteria in June 2020, (*3*) some Member States were reluctant to adopt the revised WHO recommendations. By sharing scientific evidence for time-based criteria and practices of other Member States, the Regional Office encouraged Member States to fine-tune their care pathways and/or update their protocols and practices as new evidence became available.

The Delta variant was responsible for the first major surge of reported cases that occurred in many countries in the Western Pacific Region from June 2021 (**Fig. 1**). Rapid increases in cases of severe disease needing hospitalization, cases of mild disease needing monitoring and isolation, and close contacts needing quarantine, coupled with a reduced health workforce (due to absence caused by either infection or the need to quarantine), created a tremendous strain on health systems. Inefficiencies in allocating patients to the right level of care exacerbated the problem.

**Fig. 1:**
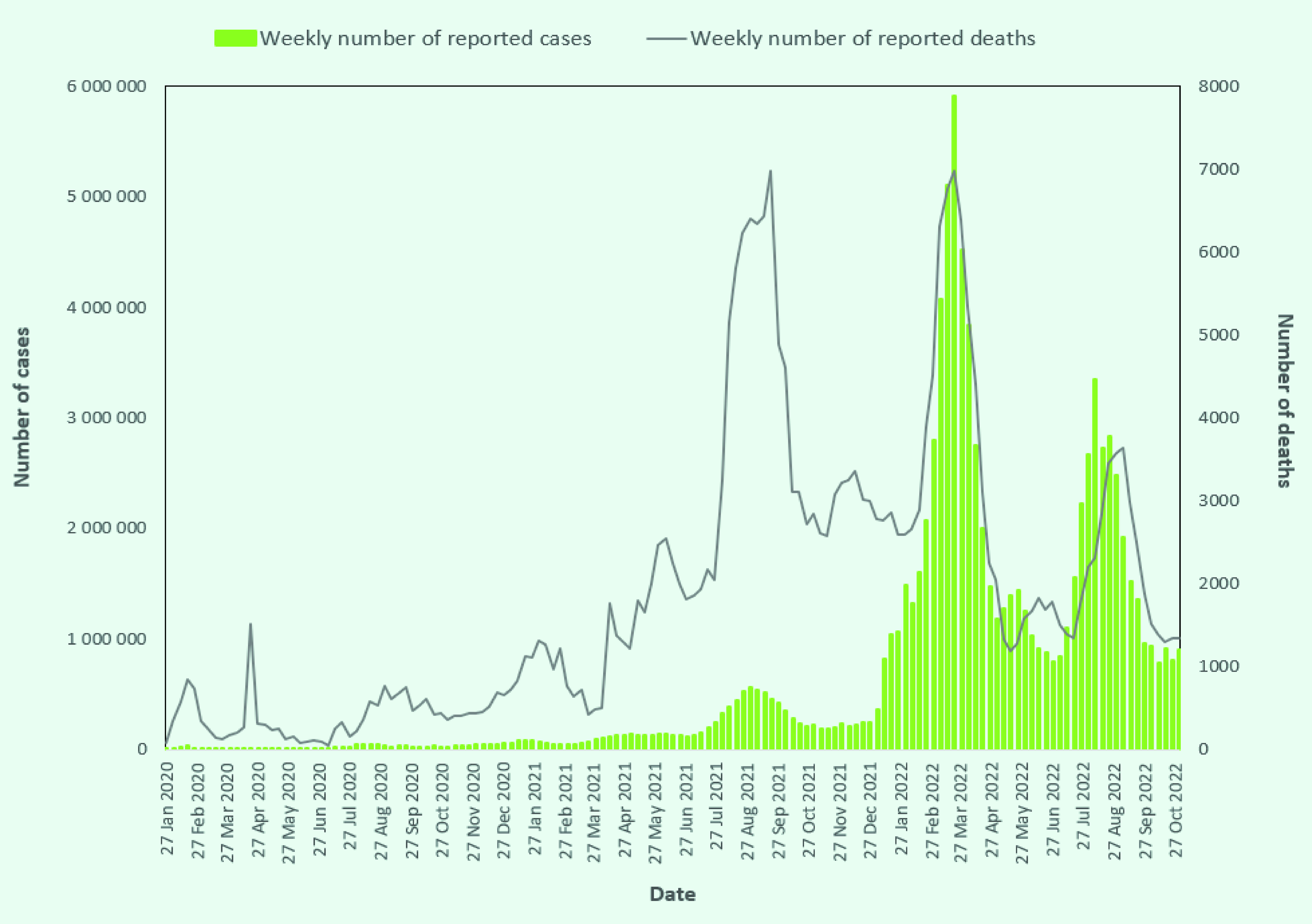
Confirmed COVID-19 cases and deaths in the Western Pacific Region, 21 January 2020 to 31 October 2022

[**insert** Figure 1]

During the surge of cases, health-care services experienced a constantly changing flow of patients as each day new patients with rapidly fluctuating medical needs entered the health-care pathway while others recovered and exited the health system. In hospital settings, intensive care unit (ICU) beds or COVID-19-designated beds had to be used for patients requiring critical care. This meant that ICU bed use, from admission to discharge, needed to be closely monitored and managed not just at the hospital level but across the local health system. In addition, patients with severe disease or with risk factors for developing severe disease required close monitoring for signs of deterioration which might necessitate admission to the critical care system. In Ulaanbaatar, Mongolia, for example, the occupancy of COVID-19-designated beds and ICU beds very quickly exceeded the available capacity in early June 2021. By monitoring the distribution of patients according to disease severity in each type of facility on a daily basis using a simple visualization system, the Ministry of Health was able to improve bed use. (*4*) This prompt action led to an immediate reduction in the number of patients waiting to be hospitalized. Similarly in the Philippines, a national surveillance system was developed to track bed utilization in all public and private hospitals in early 2020. This indicator-based system not only provided data to inform COVID-19 responses and policies, but helped avoid the overwhelming of health-care resources, showing a maximum bed utilization rate of 71.7% during the country’s Delta variant surge in mid-2021. (*5*)

At hospitals that accommodated patients with respiratory failure, oxygen capacity quickly became an urgent priority. Oxygen therapy is a cornerstone of treatment for respiratory diseases including COVID-19; however, its availability remains suboptimal in many low- and middle-income countries. Hospitals struggled not only with forecasting oxygen use and securing a sustainable supply of oxygen and consumables, but also with maintaining their oxygen system, ventilators and pulse oximeters because of the limited availability of trained biomedical engineers or similarly trained personnel. In Fiji, the situation was ameliorated by the introduction of an electronic COVID-19 clinical dashboard in mid-2021. The dashboard, which provided information not only on the availability of oxygen and its delivery devices but also on case severity, bed occupancy and management of patients isolating at home, (*6*) helped hospitals to track and forecast oxygen use in real time at the facility level. Across the Region, the WHO Regional Office supported oxygen scale-up through the procurement of ventilators, pulse oximeters and other consumables, and by training health-care workers on the use of ventilators and intensive care. The Regional Office was also instrumental in the procurement of 14 pressure swing absorption oxygen plants for 11 Member States in the Region, including eight Pacific island countries.

The pandemic called for a rapid expansion of health-care capacity. Many countries such as Viet Nam responded by establishing intermediate care facilities to accommodate patients with mild disease so that hospitals and treatment centres could focus on those with severe or critical disease. (*7*) The ability to transfer patients between facilities with different levels of medical care played a key role in facilitating this health-care pathway. Some Member States such as Japan and Singapore also established home-based care systems for those with mild disease or asymptomatic infection. (*8*, *9*)

As the pandemic progressed, the importance of being able to monitor the overall use of the health-care system became increasingly apparent. This form of situation monitoring, or “red-line analysis,” (*10*) aims to predict when health-care systems might potentially become overwhelmed by a surge in case numbers using a simple projection model and indicators such as occupancy rates of ICU beds and COVID-19 designated beds. The Regional Office supported Member States in setting up such monitoring systems. (*10*)

Throughout the pandemic, the Regional Office has supported its Member States by sharing experiences and the best available scientific evidence. This form of support was not limited to provision of information but extended to assisting countries in interpreting the available evidence, as well as formulating and implementing policies according to their local context. In this regard, the Regional Office hosted individual sessions with the governments of Cambodia, the Lao People's Democratic Republic and Mongolia, which resulted in the development of specific policies to optimize care pathways in each country.

In October 2021, after the Delta wave subsided, the focus of the Regional Office’s support and advocacy switched from pandemic response to sustained management of COVID-19. Countries were encouraged to focus effort on five key areas, as recommended by the Asia Pacific Strategy for Emerging Diseases Technical Advisory Group. The five key areas were: 1) vaccines; 2) public health and social measures; 3) health system capacity; 4) early detection and targeted response; and 5) international border measures (**Fig. 2**). (*10*) The aims of the strategy shift were to safeguard the health system from being overwhelmed; protect high-risk groups; prevent severe disease and deaths; and support social and economic recovery. Amid this effort, the Region experienced another surge of cases, starting in January 2022 and driven by the Omicron variant (**Fig. 1**). Although increased vaccination coverage across the Region helped protect vulnerable populations to some degree, the rapid increase in case numbers put pressure on health systems and resulted in increased mortality in some Member States.

**Fig. 2:**
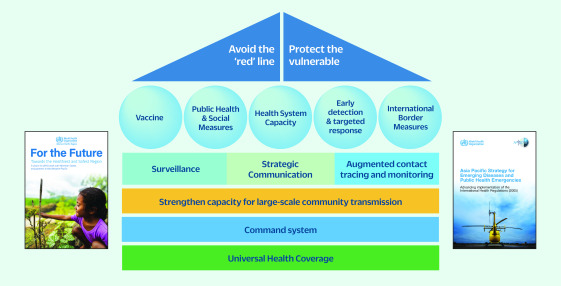
Five key areas and three supporting pillars for transitioning to sustained management of COVID-19

[**insert** Figure 2]

The Western Pacific Region has evolved a wealth of experience in COVID-19 clinical management and  health-care pathways at both national and subnational levels and across a range of economic and health system development levels. The challenges, successes and lessons shared by Member States may help countries to improve their clinical management and health-care pathways for future pandemics of respiratory infections, build robust health security preparedness capacity and move closer to universal health coverage.
